# Development of New Indonesian Propolis Extract-Loaded Self-emulsifying: Characterization, Stability and Antibacterial Activity

**DOI:** 10.34172/apb.2021.013

**Published:** 2020-11-07

**Authors:** Yandi Syukri, Annisa Fitria, Suci Hanifah, Muthiah Idrati

**Affiliations:** ^1^Department of Pharmacy, Islamic University of Indonesia, Yogyakarta 55584, Indonesia.

**Keywords:** Antibacterial, Castor oil, Propolis extract, Self-emulsifying

## Abstract

***Purpose:*** This study aimed to prepare, characterize, examine the stability and evaluation of the antibacterial activity of Indonesian propolis extract-loaded self-emulsifying (PESE).

***Methods:*** Oil, emulsifier, and co-emulsifier were selected as the carrier for the PESE formulation through a propolis-extract solubility test on each carrier, followed by evaluation of the nanoemulsion region in a pseudo ternary phase diagram. Pre-concentrate of PESE was prepared with the addition of 150 mg/mL propolis extract followed by characterization for the transmittance, globule size, zeta potential, thermodynamic stability, robustness to dilution, and accelerated stability. The selected formulation was tested for antibacterial activity using a microdilution method.

***Results:*** The PESE characterization produced a clear nanoemulsion with a globule size ranging from 13 to 45 nm and zeta potential of less than −38 mV. The PESE formulation with a composition of 150 mg/mL propolis extract, 20% castor oil, 40%–70% Kolliphor EL, and 10%–40% * polyethylene glycol* (PEG) 400 were thermodynamically stable. The PESE formulation with the composition of 20% castor oil, 40% Kolliphor EL, and 40% PEG 400 was the optimum formulation that passed the robustness to dilution evaluation and an accelerated stability test for 3 months. The antibacterial activity test on this formulation indicated improved activity against *Escherichia coli* and *Staphylococcus aureus* compared with that of propolis extract.

***Conclusion:*** These studies demonstrated that PESE in optimum formulation could be used as an antibacterial, particularly in *E. coli* and *S. aureus*.

## Introduction

Propolis or bee glue is a substance obtained from honeybees that consist of resin, wax, essential oil, and a chemical compound with a complex composition secreted by the bees, collected from tree buds and sap, and changed with an enzyme to seal open spaces in the hive. Propolis contains natural bioactive compounds, such as polyphenols, flavonoids, and caffeic acid, with its esters. These various chemical components confer abundant pharmacological activities on propolis, including antioxidant, antibacterial, anticancer, antifungal, anti-inflammatory, and antivirus effects.^1-3^

As an archipelagic country, Indonesia has a wide range of biodiversity with great potential as a source of pharmaceutical raw materials, including honeybees (*Trigona* spp. ) with their propolis. The extraction and isolation of active compounds from propolis have shown that the propolis collected from different regions in Indonesia has antibacterial and immunostimulant activities.^4,5^ A large number of publications have also highlighted the potential of propolis obtained from countries such as Chile, China, and Brazil as pharmaceutical raw material with anti-inflammatory activity,^6,7^ that from India and Thailand as an antioxidant,^1,8^ that from Argentina as an immunostimulant,^3^ and that from Brazil as an antibacterial.^9^ The ethanolic extract of propolis has also been studied and proved to have antibacterial activity, enabling its nanoemulsion preparation to be used as a food preservative.^10^ An antibacterial activity test on red propolis from Brazil also showed that propolis is effective against *Escherichia coli*,*Pseudomonas aeruginosa*, and *Staphylococcus aureus*.^11,12^

Despite the numerous studies reporting on the attractiveness of propolis as pharmaceutical raw material, propolis extract also contains active lipophilic plant secondary metabolites with poor solubility in water, which would potentially reduce the efficacy and bioavailability.^13^ One method recently developed to improve the bioavailability and efficacy of drugs is a formulation using lipid carriers to produce a self-emulsifying property that can form a clear solution when dropped in water. The dispersed active substance comprises a mixture of oil, emulsifier, and co-emulsifier in nanometer-sized particles. The application of nanoparticles for herbal preparations known as nanoherbals is suitable in Indonesia to obtain pharmaceutical raw materials from natural resources. For example, the andrographolide isolated from Indonesia’s *Andrographis paniculata* exhibits improved solubility and bioavailability when in a self-nano-emulsifying formulation.^14^

Novel delivery systems for poorly water-soluble active compounds from natural resources have recently drawn more attention as the development of such systems could improve the solubility and bioavailability of active substances. Against this background, this study was established to the formulation and characterize of Indonesian propolis extract-loaded self-emulsifying (PESE) as an antibacterial agent.

## Materials and Methods

### Materials

Propolis extract was obtained from Bee House (Surabaya, Indonesia), Labrafac and Labrasol were purchased from Gattefose (Saint-Priest, France), Cremophor RH 40, Kollisolv, and Kolliphor were obtained from BASF (Jakarta, Indonesia), castor oil, sunflower oil, sesame oil, virgin coconut oil (VCO), Tween 20, polyethylene glycol (PEG) 400, and propylene glycol were purchased from Brataco (Yogyakarta, Indonesia); *Staphylococcus aureus* ATCC 25923,*Salmonella typhimurium* ATCC 14028,*Salmonella typhi* ATCC 35664, *Escherichia coli* ATCC 8739, Mueller Hinton Agar (Oxoid) and Mueller Hinton Broth (Oxoid), and 0.5 McFarland Standard were obtained from ATCC (Virginia, USA), dimethyl sulfoxide (DMSO), sterile solution of 0.9% NaCl and MTT were purchased from Sigma-Aldrich (Singapore).

### Pre-formulation study for self-emulsifying system

Oil, emulsifier, and co-emulsifier as carriers were selected based on their ability to provide the highest solubility for propolis extract. A total of 100 mg of propolis extract was dissolved in each carrier, beginning with the lowest volume sequentially from 0.1, 0.25, 0.5, 0.75, 1.0, 1.25, to 1.5 mL. Carriers with the smallest amount but most significant ability to dissolve propolis extract became the chosen carriers.

A pseudo ternary phase diagram for the selected oil, emulsifier, and co-emulsifier obtained through a solubility test without the addition of propolis extract was constructed by plotting the carriers in a pseudoternary phase diagram to identify the nanoemulsion region of three-carrier combinations. A total of 20 compositions from oil, emulsifier, and co-emulsifier were prepared for the construction of this diagram with ranges of oil, emulsifier, and co-emulsifier phase concentrations of 10%–50%, 10%–80%, and 10%–40%, respectively. The formulation design for the construction of a pseudoternary phase diagram is presented in Table 1. Nanoemulsion formation for the compositions in Table 1 was identified after 100-fold dilution using double-distilled water, followed by measurement of the clarity based on the transmittance value using a UV-vis spectrophotometer (Shimadzu UV 1800, Japan) at a wavelength of 650 nm. Diluted carrier mixtures with more than 80% transmittance were categorized as transparent and plotted in the ternary phase diagram of oil, emulsifier, and co-emulsifier.^14^

**Table 1:** Formulation design for the construction of pseudo ternary phase diagram

**Formulation**	**Oil: Smix**	**Oil** **(%)**	**Emulsifier** **(%)**	**Co-emulsifier (%)**
F1	1: 9	10	80	10
F2		10	70	20
F3		10	60	30
F4		10	50	40
F5	2: 8	20	70	10
F6		20	60	20
F7		20	50	30
F8		20	40	40
F9	3: 7	30	60	10
F10		30	50	20
F11		30	40	30
F12		30	30	40
F13	4: 6	40	50	10
F14		40	40	20
F15		40	30	30
F16		40	20	40
F17	5: 5	50	40	10
F18		50	30	20
F19		50	20	30
F20		50	10	40

### Formulation of propolis extract-loaded self-emulsifying

PESE as pre-concentrate was prepared by adding 150 mg/mL propolis extract into the mixture of oil, emulsifier, and co-emulsifier until a clear solution was obtained. This pre-concentrate was then stored at ambient temperature for further studies.

### Characterization of self-emulsifying propolis extract

#### Percentage transmittance

The transmittance value was determined by diluting PESE 100-fold in double-distilled water as a blank and measuring the percentage transmittance using a UV-vis spectrophotometer (Shimadzu UV 1800, Japan) at a wavelength of 650 nm.

#### Globule size and zeta potential

PESE was diluted 100-fold in double-distilled water and measured for the globule size and zeta potential of dispersed particles using a laser dynamic light scattering (DLS) method in a particle size analyzer designed explicitly for measuring nanometer-sized particles (Horiba SZ 100, Japan).

### Thermodynamic stability studies

#### Centrifugation

PESE was diluted 25 times and centrifuged at 3500 rpm for 15 min to examine the precipitation and phase separation.

#### Heating–cooling cycle

PESE was diluted 25 times and stored at 4°C for 8 h, followed by storage at 45°C for 8 h. The cycle was repeated six times in 48 h. After the last cycle, the dilution was centrifuged for 15 min at 3500 rpm, and the precipitation and phase separation were examined.

#### Freeze-thaw cycle (accelerated aging)

After 25-fold dilution, PESE was stored at −40°C for 8 h, followed by storage at ambient temperature (25°C) for 8 h with six cycles in 48 h. The dilution was centrifuged for 15 min at 3500 rpm to examine the precipitation and phase separation.

### Robustness to dilution

PESE was tested with a multiple-dilution process to resemble in vivo conditions, in which a formulation would be gradually diluted 25, 50, 100, and 250 times. The globule size in nanoemulsions was then identified to guarantee a stable size after multiple dilutions.

### Accelerated stability studies

The formulations passing robustness to dilution test were then subjected to an accelerated stability test in a climatic chamber under storage conditions of 40°C ± 2°C/75% RH ± 5% RH for 3 months. The percentage transmittance and globule size were then measured in the first, second, and third months.

### Antibacterial activity

The minimum inhibition concentration (MIC) and minimum bactericidal concentration (MBC) of PESE, propolis extract, and placebo were determined using a microdilution method from the Clinical Laboratory Standards Institute (CLSI).^15^ The sample was diluted using Mueller-Hinton Broth (MHB) to obtain a series of concentrations ranging from 1 to 0.012 mg/mL with a volume of 100 μL for each well of the microplate. As much as 10 μL of inoculum with 10^6^ CFU/mL bacteria was put into each well. The test employed control bacteria in a well containing MHB and dimethyl sulfoxide (DMSO) 10% with test bacteria as well as a medium control in a well containing MHB without bacterial inoculum. The culture was incubated aerobically at 37°C for 24 h. MIC is defined as the extract concentration at which no bacterial growth occurs (clear solution) as opposed to the bacterial control. Samples from several wells expected to have MIC were then cultured on Mueller-Hinton Agar (MHA) to identify MBC. The value of MBC can be determined if no live bacterial colonies are found in the agar medium. In addition to MIC and MBC, the percentage of bacterial cell death was also determined using MTT assay. The MTT assay reflects the number of viable cells present in well. The enzymes of viable cells are capable of reducing the tetrazolium dye of MTT, which has a yellow color, to its soluble formazan, which has a purple color. For this, after MTT reagent had been added to each well, the percentage of dead cells was calculated by reading the absorbance value of each sample using a microplate reader at a wavelength of 570 nm plotted into the following equation:

$$Percentage\;of\;cell\;death=\frac{Absorbance\;of\;bacterial\;control-Absorbance\;of\;sample}{Absorbance\;of\;bacterial\;control}\times 100\%$$

### Statistical analysis 

ANOVA and *t* test assessed the differences in antibacterial activity between placebo, propolis extract, and PESE. The variations of the averages are shown as SD, and differences were considered significant at *P*  < 0.05.

## Results and Discussion

### Pre-formulation study for self-emulsifying system

**Figure 1:**
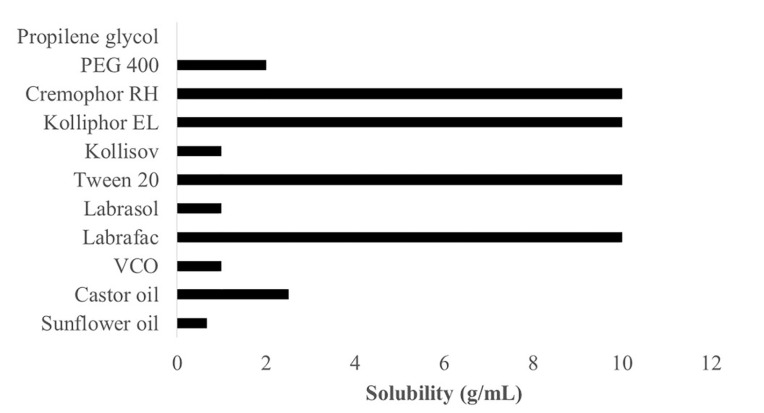
Solubility study of propolis extract in various oil, emulsifier and co-emulsifier (n=3).

A test of propolis extract solubility in carriers is essential to obtain a stable formulation. It is required to guarantee that the formed nanoemulsion does not precipitate out in the digestive tract.^16^ The generally recognized as safe category has become the main criterion to consider when selecting emulsifier and co-emulsifier since these materials have to be pharmaceutically acceptable for oral administration. Another consideration is that the required hydrophilic-lipophilic balance (HLB) value to form o/w emulsion has to be more than 10.^17^ The results of solubility tests for propolis extracts with carriers comprising oil, emulsifier, and co-emulsifier are presented in Figure 1.

Figure 1 shows that propolis extract has the best solubility in the order of castor oil (2.5 g/mL), VCO (1 g/mL), and sunflower oil (0.67 g/mL). Castor oil has an HLB value of 14, which is higher than those of coconut oil (HLB: 8) and sunflower oil (HLB: 7), indicating that a higher HLB in carriers enables better dissolution of propolis extract.^18^ Propolis extract has the best solubility in Cremophor RH 40, Kolliphor, Tween 20, and Labrafac as emulsifiers, at 10 g/mL. Meanwhile, the ability to dissolve in Kollisolv and Labrasol emulsifiers is 1 g/mL, and PEG 400 as a co-emulsifier can dissolve propolis at 2 g/mL. Still propolis does not dissolve in propylene glycol co-emulsifier.

**Figure 2:**
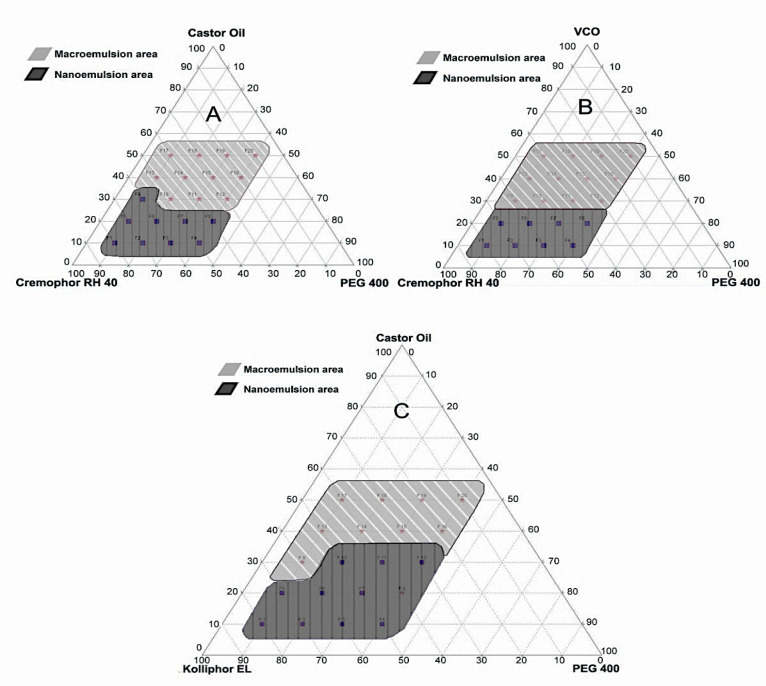
Pseudo ternary phase diagram indicating the region of o/w nanoemulsion and macroemulsion formations with castor oil, Cremophor RH 40, and PEG 400 (A); VCO, Cremophor RH 40, and PEG 400 (B); and castor oil, Kolliphor EL, and PEG 400 (C) as the carriers

Given these findings, the selected oil phase consisted of castor oil and VCO, while Cremophor RH 40 and Kolliphor EL were selected as emulsifiers, and PEG 400 was selected as a co-emulsifier. A pseudoternary phase diagram was constructed to identify the nanoemulsion region of the formulations prepared to determine the ideal phase among the chosen oils, emulsifiers, and co-emulsifiers.^19^ This diagram is intended to facilitate the determination of the region, including the self-emulsifying region and macroemulsion region, and also to the identification of the best composition for drug loading. Only the most appropriate combination of oil, emulsifier, and co-emulsifier with accurate concentrations can produce self-emulsifying when diluted with water. The phase compositions of each oil, emulsifier, and co-emulsifier with the ability to self-emulsify when dropped in water to form a nanoemulsion are presented in the pseudoternary phase diagram in Figure 2.

Figure 2 shows that the combination of castor oil, Cremophor RH 40, and PEG 400 carrier has a phase composition of 10%–30% oil, 40%–80% emulsifier, and 10%–40% co-emulsifier. The phase ranges of VCO, Cremophor RH 40, and PEG 400 are 10%–20% oil, 40%–80% emulsifier, and 10%–40% co-emulsifier, while the combination of castor oil, Kolliphor EL, and PEG 400 is composed of 10%–30% oil, 30%–80% emulsifier, and 10%–40% co-emulsifier. Therefore, the composition of castor oil (oil), Kolliphor EL (emulsifier), and PEG 400 (co-emulsifier) was selected as the carrier for the formulation of self-emulsifying for propolis extract preparation. The carriers of a self-emulsifying formulation consisting of oil, emulsifier, and co-emulsifier must have a high solubilizing capacity for the selected drugs to achieve optimum drug loading.^20-22^

### Propolis extract-loaded self-emulsifying formulation

Propolis extract must dissolve in carriers, marked by the absence of phase separation, color change, and precipitation for 48 h, which would prove that the extract is stable in the formulation. PESE as pre-concentrate was made with 150 mg/mL drug loading followed by the characterization that includes determination of the transmittance value, globule size, and zeta potential, as shown in Table 2.

**Table 2:** The effect of different concentrations of oil, emulsifier and co-emulsifier on the particle size, zeta potential, transmittance, description, and thermodynamic stability test of PESE (n=3)

**Formulation**	**Globule size (nm)**	**Zeta potential (mV)**	**Transmittance (%)**	**Description**	**Centrifugation**	**Heating cooling cycle**	**Freeze thaw cycle**
F1	17.3±0.3	-38.2±0.6	99.16±0.02	Clear	Stable	Stable	Stable
F2	14.3±0.2	-41.7±0.2	98.61±0.01	Clear	Stable	Stable	Unstable
F3	11.7±0.2	-42.2±0.9	99.47±0.02	Clear	Stable	Stable	Stable
F4	20.9±0.1	-41.3±1.5	99.42±0.04	Clear	Stable	Stable	Stable
F5	13.8±0.3	-35.7±3.5	99.20±0.01	Clear	Stable	Stable	Stable
F6	16.3±0.3	-44.8±1.4	96.32±0.03	Clear	Stable	Stable	Stable
F7	15.5±0.4	-40.2±0.3	93.49±0.11	Clear	Stable	Stable	Stable
F8	30.5±0.5	-35.4±0.9	94.57±0.90	Clear	Stable	Stable	Stable
F9	20.8±0.5	-38.0±1.6	95.78±0.01	Clear	Stable	Stable	Stable
F10	27.3±0.1	-40.5±1.3	91.30±0.01	Clear	Stable	Stable	Stable
F11	44.7±0.8	-41.5±0.4	83.14±0.01	Clear	Stable	Stable	Stable
F12	NA	NA	51.95±0.01	Cloudy	NA	NA	NA
F13	NA	NA	36.69±0.01	Cloudy	NA	NA	NA
F14	NA	NA	42.16±0.11	Cloudy	NA	NA	NA
F15	NA	NA	0.38±0.01	Cloudy	NA	NA	NA
F16	NA	NA	58.91±0.16	Cloudy	NA	NA	NA
F17	NA	NA	0.28±0.00	Cloudy	NA	NA	NA
F18	NA	NA	0.52±0.00	Cloudy	NA	NA	NA
F19	NA	NA	2.44±0.02	Cloudy	NA	NA	NA
F20	NA	NA	15.15±0.02	Cloudy	NA	NA	NA

NA : Not Available. It mean particle size was not detected. The variations of the averages are shown as SD.

#### Transmittance

Transmittance was measured to determine the clarity of PESE dilution compared with that of double-distilled water. The required percentage transmittance for self-emulsifying is above 80% or approximately 100%, and a transmittance rate approaching 100% means that the globule size has reached the nanoparticle range.^23^

Table 2 shows that formulation 1 up to formulation 11 have more than 80% transmittance and can thus be described as clear solutions. Therefore, these formulations were selected as fulfilling the requirement of spontaneously dispersed transparent emulsions to form nanoemulsion. The clarity is assessed to identify the efficiency of a formation of a clear self-emulsifying by determining whether the dispersion reaches equilibrium in a short time and a reproducible manner. Nanoemulsion with a globule size of less than 100 nm is generally transparent, and the higher the percentage transmittance. The compatibility of selected carriers and the isotropic nature of a formulation for preliminary study are usually determined by identifying the transmittance.^24,25^

#### Globule size

In the formulation of self-emulsifying preparation, globule size is one of the critical factors to consider. It is one of the main properties of a nanoemulsion preparation as it is the crucial factor for improving the solubility and bioavailability of an active substance.^26^ Globule size was identified for 11 selected formulations under the nanoemulsion region of the pseudoternary phase diagram with an acceptance criterion for this variable of less than100 nm. Table 2 shows that all of the 11 formulations have an acceptable globule size of below 50 nm. Increased globule size was indicated by increased oil concentration, such as in formulation 8 (F8) up to formulation 11 (F11), where globule size increased along with the increase in oil concentration (20%–30%). This finding is supported by another result showing an increased globule size due to reduced optical clarity marked by a decrease in transmittance.

A high concentration of emulsifier in a formulation can improve the ability to reduce globule size, but an emulsifier at a high concentration may cause stomach irritation.^27^ Therefore, a formulation with lower emulsifier concentration, good clarity, and appropriate globule size is considered ideal. However, this approach has several disadvantages, including in the range of globule size between 0.2 nm and 2 μm in the DLS method, with findings showing that this technique is unable to detect any microparticles. This method contrasts with the laser diffraction with the ability to measure particles ranging between 20 nm and 2 mm and can lead to underestimation of the number of small nanosized particles..^28^ These data are indicated by F12–F20 in Table 2.

#### Zeta potential

Zeta potential is as essential as an optimized globule size since both can affect self-emulsifying stability. Zeta potential of electrokinetic potential is defined as the electric potential created by charge separation in the liquid-liquid interface of a double layer. This phenomenon influences the energy of interaction between particles that controls the stability of particles and drug delivery systems. Besides, zeta potential can be associated with flow properties. Therefore, zeta potential and globule size are measured to select an ideal composition based on several comparison possibilities.^29^

It is crucial to determine the zeta potential of PESE to identify the oil droplet charge in an emulsion. The increasing electrostatic charge between globules can prevent particle coalescence, and conversely, a reduction in the electrostatic charge can prevent phase separation.^30^ Since a zeta potential of ±30 mV is generally suitable for system stability, the formulation should be optimized to fulfill this requirement. The globule charge of the oil phase is negative due to the presence of emulsifier and co-emulsifier in a formulation.^31^

Table 2 shows that all of the formulations have a zeta potential of less than −30 mV. Zeta potential formed in a transparent emulsion is believed to affect bioavailability because of the fatty acid in the carrier structure. A negative value indicates the negative charge of preparation, and sufficient repulsion among emulsion droplets existed to form an un-coagulated system and, therefore, means a stable system.^32^ Zeta potential of lower than −30 mV in precise emulsion results in a stable, durable formulation. If the zeta potential is low, the force of attraction will increase, thus forming an aggregate that will break the emulsion system.

### Thermodynamic stability studies

Through in situ solubilization, self-emulsifying produces nanoemulsions that have excellent stability against creaming, cracking and precipitation. Different self-emulsifying formulations, however, generally begin to precipitate out during prolonged storage. It is necessary to conduct thermodynamic stability studies to examine the durability of self-emulsifying formulations under such conditions.^24^

Thermodynamic stability studies are specifically required to test the stability of PESE preparations against extreme conditions, thus providing a guarantee of the stability and durability of PESE. PESE becomes dispersed when exposed to the digestive fluid to form a transparent emulsion system. This emulsion must withstand precipitation and phase separation over a long period. The results of thermodynamic stability studies are presented in Table 2.

PESE formulations that fulfill the characterization parameters were subjected to thermodynamic stability tests, including centrifugation. These tests were performed to assess the strength of PESE preparations exposed to vigorous kinetic motion, revealing the preparation stability after prolong storage.^23^

A development must withstand phase separation or precipitation after 1:25 dilution and 30 min of centrifugation at 4000 rpm. As shown in Table 2, formulation 1 (F1) up to formulation 11 (F11) exhibited the absence of both phase separation and precipitation with clear dilution results. Therefore, F1 to F11 passed the centrifugation test and could proceed to the heating-cooling stability test.

This latter test was applied to determine the stability of PESE preparations under conditions of changing temperature. Table 2 showed that all of the formulations passed the heating–cooling test, indicated by the absence of precipitation and phase separation.

Kinetic strength is one of the required characteristics of PESE because stability in this regard can distinguish a nanoemulsion from an emulsion. PESE has to be able to form nanoemulsions spontaneously when dissolved in a solvent without exhibiting phase separation as well as precipitation during storage.^33^ The formulations passing the heating-cooling cycles were then tested in freeze-thaw cycles.

Table 2 indicates that all of the formulations passed the freeze-thaw test since they did not show any phase separation or precipitation during the six cycles of freeze-thaw. As part of thermodynamic stability studies, this test has an identical aim to the heating-cooling test, but with a difference in storage temperatures. While heating and cooling cycles are performed at 40°C and 4°C, freeze and thaw cycles take place at 25°C and −20°C.^34^

### Robustness to dilution

Robustness to dilution is measured to ensure that in vivo drug precipitation does not occur at any diluting conditions that can influence drug absorption. Formulations are diluted several times in various media to imitate in vivo conditions, as well as to guarantee the uniformity of nanoemulsion formation.^35^

To resemble in vivo conditions, PESE underwent a process of multiple dilutions of 25, 50, 100, and 250 times. It was essential to guarantee emulsion uniformity after the dilution process. Formulations passing the stability test were subjected to the robustness to dilution test to examine whether uniform emulsions spontaneously formed at different dilution rates. The results of the robustness to dilution test are presented in Table 3.

**Table 3:** Robustness to dilution test of PESE (n=3)

**Formulation**	**Globule size (nm)**			
	**1:25**	**1:50**	**1:100**	**1:250**
F1	14.2±0.6	13.3±0.6	17.3±0.3	124.8±2.5
F2	109.6±2.8	9.5±0.1	14.3±0.2	104.6±0.5
F3	16.7±0.9	11.2±0.2	11.7±0.1	53.3±1.9
F4	21.5±0.1	16.1±0.1	20.8±0.1	75.3±0.6
F5	11.5±0.1	14.4±0.2	13.8±0.3	16.3±0.4
F6	12.2±0.2	12.3±0.1	16.2±0.3	18.1±0.1
F7	13.5±0.6	15.7±0.1	15.5±0.4	17.6±0.2
F8	35.2±0.6	25.4±0.2	30.5±0.5	33.3±0.4
F9	17.4±0.3	43.1±0.2	20.7±0.5	40.9±0.3
F10	61.6±0.1	23.2±0.2	27.3±0.1	24.3±0.2
F11	118.3±0.1	116.1±0.4	44.6±0.8	58.4±0.7

The variations of the averages are shown as SD.

Robustness to dilution was assessed of the globule size. Then the data were compared for all of the four dilutions. A formulation must maintain a globule size below 200 nm and similar size or insignificant size changes among globules after the different dilutions to prove the robustness.

Of the 11 formulations, all had a globule size smaller than 200 nm, but the best formulations with the most stable size and insignificant change were F5, F6, F7, and F8. Meanwhile, F1 up to F4 had highly significant differences in globule size between dilutions 1:25 and 1:50 or between dilutions 1:50 and 1:250. For F9, F10, and F11, the globule size also changed significantly and visibly, as these three formulations appeared cloudy. Therefore, only F5 to F8 proceeded to the subsequent tests.

The findings of an unstable globule size were probably caused by the use of Kolliphor EL as an emulsifier, which can form aggregates and induce coagulation upon dilution at a certain rate. Indeed, a study of Kolliphor’s stability in the gastrointestinal (GI) tract has proved that Kolliphor in a formulation cannot be quickly and easily dissolved and digested in GI fluid.^36^ Therefore, in several compositions for preparations containing Kolliphor EL with different dilutions, Kolliphor EL may have imperfectly dissolved, resulting in large and inconsistent globule sizes.

### Accelerated stability studies

These tests were conducted to prove the ability of a preparation to remain stable during storage. The results of the accelerated stability tests for PESE are presented in Table 4. Table 4 showed that the globule sizes of F5 to F8 in the first, second, and third months remained stable as a nanoparticle. The globule size changed from 15.8 to 37.9 nm. The polydispersity index (PDI) indicates a narrow particle size distribution. The PDI of nanoparticle changed from 0.11 to 0.29.^37,38^

**Table 4:** Accelerated stability test of PESE (n=3)

**Formulation**	**Month**	**Transmittance (%)**	**Globule size (nm)**	**PDI**
F5	1	98.39±0.10	15.8±0.1	0.11±0.08
	2	97.98±0.02	20.6±0.4	0.29±0.07
	3	97.28±0.01	12.4±0,1	0.27±0.06
F6	1	98.53±0.06	17.3±0.1	0.15±0.08
	2	97.50±0.08	18.1±0.1	0.21±0.08
	3	97.01±0.02	15.9±0.2	0.17±0.04
F7	1	97.81±0.10	18.7±0.1	0.29±0.09
	2	97.80±0.01	19.0±0.2	0.25±0.02
	3	97.52±0.02	18.9±0.2	0.16±0.06
F8	1	98.26±0.25	37.9±0.2	0.28±0.07
	2	96.57±0.02	32.1±0.8	0.14±0.07
	3	94.45±0.01	32.2±0.2	0.24±0.02

The variations of the averages are shown as SD.

**Figure 3:**
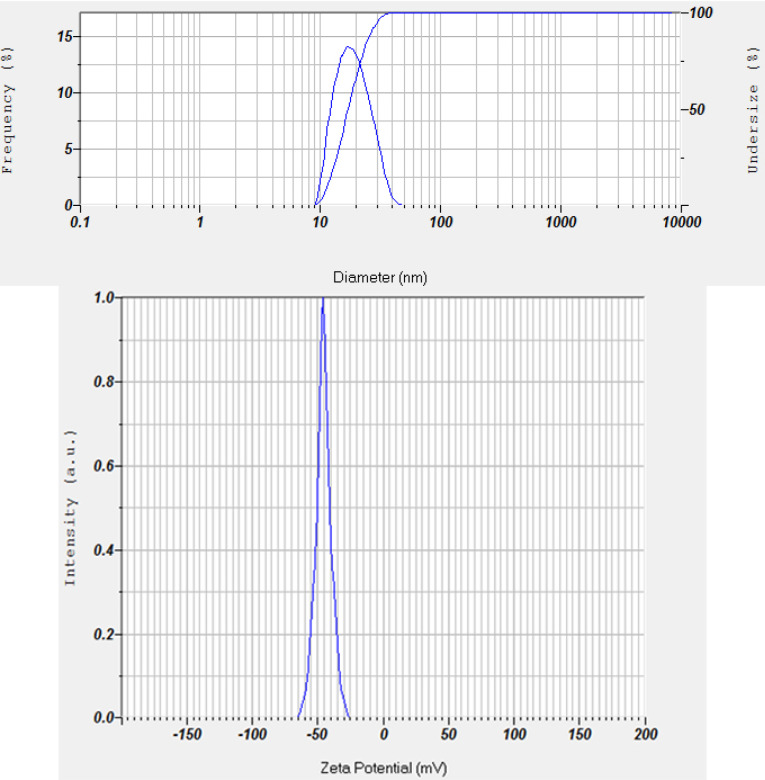
Particle size distribution (top) and zeta potential (bottom) of the optimum PESE formulation (100 times diluted with double distilled water)

**Figure 4:**
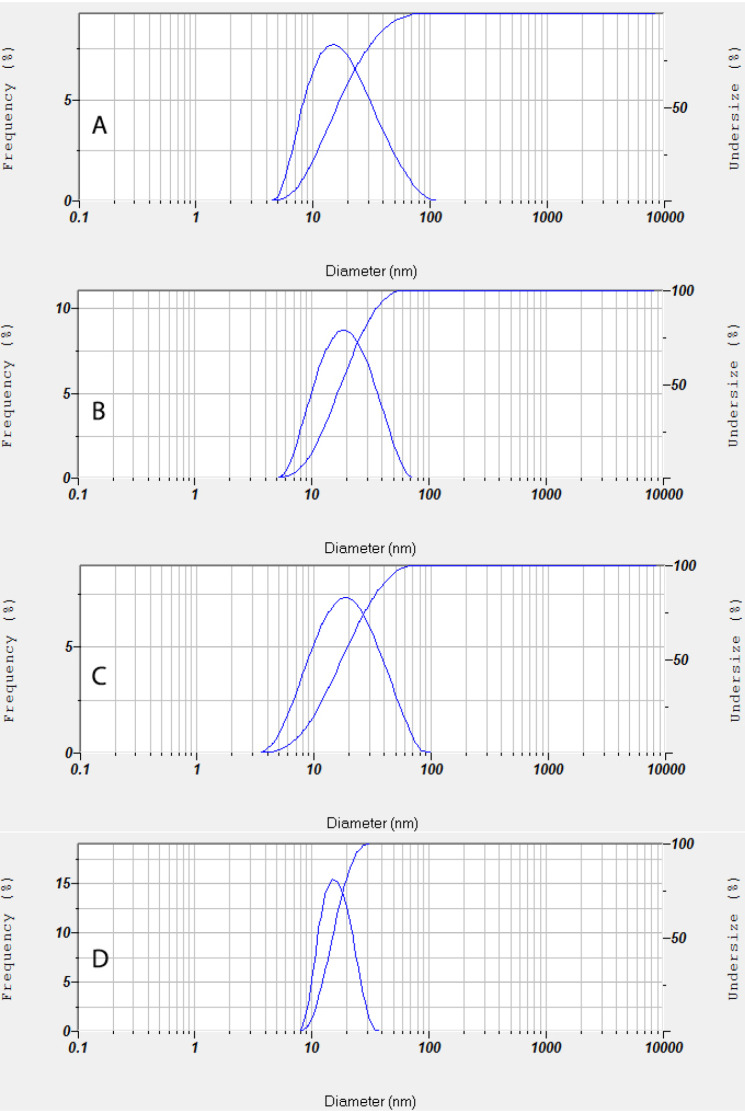
Particle size distribution of the optimum PESE formulation (100 times diluted with double distilled water) during accelerated stability test; (A) before storage, (B) storage after 1 month, (C) storage after 2 months, and (D) storage after 3 months.

The accelerated stability test evaluation showed that the PESE formulation with the composition of 20% castor oil, 40% Kolliphor EL, and 40% PEG 400 was the optimum formulation. The particle size distribution and zeta potential of the optimum PESE formulation were presented in Figure 3. This optimum formulation also demonstrated stable the particle size before storage, storage after 1 month, storage after 2 months, and storage after 3 months, as shown in Figure 4. These findings are also supported by the transmittance rates of more than 80%, indicating that these four formulations remained as clear solutions during the 3-month storage period.

### Antibacterial activity

An antibacterial activity test was performed to identify the activity of PESE against several bacteria based on the values of MIC and MBC. Other than MIC and MBC, the percentage of cell death was also measured to examine the ability of PESE preparation to kill bacteria upon exposure at a specific concentration based on the obtained MIC. The activity parameters were assessed at a concentration of 150 mg/mL as the dose of propolis extract in PESE formulation with equivalent 0.293 mg/mL of propolis. The results of this antibacterial activity test are presented in Table 5.

**Table 5:** Antibacterial activity test for placebo, propolis extract and PESE formulation (n=3)

**Parameters**	***Escherichia coli ATCC *** **8739**			***Pseudomonas aeruginosa *** **9027**			***Staphylococcus aureus *** **25923**			***Salmonella typhimurium *** **14028**		
	**Placebo**	**Propolis extract**	**PESE**	**Placebo**	**Propolis extract**	**PESE**	**Placebo**	**Propolis extract**	**PESE**	**Placebo**	**Propolis extract**	**PESE**
MIC (mg/mL)	1.16	NA	1.17	NA	NA	NA	4.65	4.68	2.34	NA	NA	NA
MBC (mg/mL)	2.33	NA	2.34	NA	NA	NA	9.35	9.37	4.68	NA	NA	NA
Percentage of cell death (%)	47.50	NA	56.67	NA	NA	NA	48.26	30.96	57.36	NA	NA	NA

NA: Not Active, Placebo: Formulation contains only the vehicle.

The MIC test showed that PESE had activity against bacteria, with MIC ranging from 1 to 2.5 mg/mL. This data meets the criterion in a previous study suggesting that an extract of natural ingredients can be categorized as having high inhibitory activity against bacterial growth if the MIC value is approximately 1 mg/mL.^39^ In contrast, the propolis extract exposed to bacteria at the highest concentration (150 mg/mL) exhibited no antibacterial activity, except against S. aureus at 4.68 mg/mL. This finding is supported by some studies describing that the MIC range of 1–10 mg/mL for propolis originating from different regions is associated with antibacterial activity against *S. aureus*. Another study suggested that the antibacterial activity of propolis against gram-positive bacteria such as *S. aureus* is higher than that against gram-negative ones.^40-42^

This variety is due to differences in the composition of cell walls of gram-positive and gram-negative bacteria, which affect compound penetration. All of the bacteria used in this study, *E. coli, P. aeruginosa*, and *Salmonella typhimurium*, are from the gram-negative group, which has more complex cell walls than the gram-positive group. Consequently, the antibacterial activity against this group is lower, particularly the activity of antibacterial compounds that are poorly water-soluble.^43^

The differences in MIC values between propolis extract and PESE preparation indicate that PESE can provide better activity than the extract form. This finding is supported by an assessment of the ability to kill bacteria that results in a higher percentage of cell death of PESE as opposed to the extract. Exposure to bacteria with a concentration of MIC in PESE can kill more than 50% of the total live bacterial cells. It was also found that PESE is active against Gram-negative bacteria compared with propolis extract, although the activity is limited to certain species of bacteria.

Antibacterial activity is mainly affected by the ability of a compound to penetrate the cell membrane and cell wall of bacteria. This study demonstrated that PESE has a better penetration ability than the extract. The ability of PESE to form emulsions and maintain physical and chemical stability dominantly affects the antibacterial activity provide.^44^

## Conclusion

Propolis extract can be formed into a self-emulsifying preparation with a composition of 150 mg/mL propolis extract, 20% castor oil, 40%–70% Kolliphor EL, and 10%–40% PEG 400. This formulation provides ideal characteristics and stability with more than 80% transmittance, 11–45 nm globule size, −35 mV to −45 mV zeta potential, and a clear solution without phase separation or precipitation. This optimum formulation was also found to be stable during 3 months of storage in an accelerated stability test, and its antibacterial activity against *E. coli* and *S. aureus* was higher than that of propolis extract.

## Ethical Issues

There are no ethical issues.

## Conflict of Interest

The authors declare no conflicts of interest in this study.

## Acknowledgments

The authors would like to thank the Minster of Research and Higher Education of Republic of Indonesia for funding this work (grant numbers: 042/DirDPPM/70/DPPM/PTUPT-KEMRISTEKDIKTI/II/2018).
